# Load Balancing Cloud Storage Data Distribution Strategy of Internet of Things Terminal Nodes considering Access Cost

**DOI:** 10.1155/2022/7849726

**Published:** 2022-01-24

**Authors:** Jiansheng Wu, Weimin Xu, Jiarong Xia

**Affiliations:** ^1^China Tobacco Zhejiang Industrial Co., Ltd.,, Ningbo, Zhejiang 315504, China; ^2^School of Mathmatics, Hangzhou Normal University, Hangzhou 311121, China

## Abstract

With the rapid development of Internet of Things (IoT) technology, IoT terminal nodes are facing many challenges in data storage, distribution, and data management. In particular, in the IoT terminal nodes considering access cost, the corresponding data distribution and storage are professional, complex, and miscellaneous. Based on the abovementioned current situation, this article innovatively proposes a complex sensor data placement algorithm based on the cloud storage distribution of IoT terminal nodes. Under this algorithm, the accurate division of IoT data I/O methods is realized through reasonable configuration. Through the adaptive sensing algorithm, while fully considering the access cost of the algorithm, the performance of the IoT data storage system is further optimized. In the corresponding terminal node load balancing problem, this article innovatively proposes the terminal node data sorting and distribution algorithm through the node data. The sorting and distribution algorithm realizes the precise segmentation of the IoT data to be processed, thereby realizing the improvement of data reading and processing speed. Based on the proposed algorithm, this article designs a load balancing cloud storage data distribution optimization system of IoT terminal nodes considering access cost and carries out experimental verification in a real environment. The experimental results show that the data pattern division accuracy corresponding to the proposed distribution strategy is improved to 97.13% and the corresponding data access efficiency is improved to 98.3%, compared with the traditional distribution strategy. Therefore, the data distribution strategy proposed in this article has obvious performance advantages and further promotion value.

## 1. Introduction

The main architecture of the Internet of Things (IoT) includes the perception layer, network layer, management layer and application layer. The conventional sensing layer is mainly used to collect relevant physical information, such as temperature, humidity, air composition, and optical signals. The continuous collection and storage of data by the relevant sensor networks of the IoT cause problems of data distribution and data management [1–3]. The relevant data of the IoT are mainly classified into structured data and unstructured data. The corresponding structured data mainly depend on open-source databases and other products, while the storage of the corresponding unstructured data depends on the database software developed by the corresponding unstructured data model. At the data storage level of the IoT, cloud storage technology, as the development direction of data storage, mainly processes and analyzes a large number of different types of data generated by the IoT through cluster application, network technology, and distributed storage technology and provides external data access and storage [4, 5]. The conventional cloud storage architecture includes the application interface layer, infrastructure layer, and storage layer. The corresponding application interface layer mainly includes network access, user authentication, permission management, public API interface, application software interface, and network service interface. Moreover, the corresponding infrastructure layer includes the cluster system, distributed file system, grid system, content distribution and data deduplication, data compression, and data encryption processing. The corresponding storage layer mainly includes storage virtualization, centralized storage management, status monitoring, maintenance, and upgrading [6]. Data placement and node load balancing are important technologies of distributed storage in cloud storage technology. Load balancing mainly realizes the dynamic expansion of storage resources to further improve the utilization of resources, minimize the data response time, and further reduce the fault recovery and data switching time to ensure the quality of user data access in the IoT system [7, 8]. At the level of corresponding data placement and distribution, the flat distribution of the data system is of great significance. The directory organization corresponding to the traditional data distribution is unreasonable, and the node data distribution is too scattered or too centralized, which will further affect the response-ability of the data system, further reduce the system performance, and increase the data maintenance cost [9, 10]. Compared with other storage models, cloud storage has the following advantages: (1) easy to expand: the storage space is expanded in time according to the number of users and space of the server, which will also not affect the use of the front-end users; (2) reliable and safe: data synchronization effectively avoids media storage of data. Cloud storage can fully realize load balancing, which can realize the flow distribution control service of distributing access traffic to multiple back-end cloud servers according to the forwarding strategy. Moreover, the external service capabilities of the application system are extended through traffic distribution, and the availability of the application system is improved by eliminating single points of failure.

The traditional cloud storage data distribution strategy of load balancing of IoT terminal nodes has some disadvantages in data placement and load balancing technology. At the data placement level, the disadvantages are as follows: differences in data storage performance and availability storage cost between different data storage service providers and differences in different data reading and writing modes in the IoT sensor network. Therefore, the traditional data placement algorithm only meets a single optimization goal and cannot meet the application storage requirements under the huge amount of data in IoT. Based on this, constructing an efficient data placement algorithm to optimize the performance of different data access is of great significance to improve the performance of cloud storage data systems for load balancing of IoT terminals [11, 12]. For the problem of load balancing, after the corresponding IoT data are initialized, as the storage system continues to serve various big data-related applications on the upper layer, the data layout corresponding to each cloud storage system will be unevenly distributed, which will lead to a serious decline in the performance of data access [13–15]. After the storage system has been running for a period of time, it will become normal that the corresponding data load is seriously unbalanced. Once the corresponding load of a data storage service provider is very high, the system may be blocked and stagnant. When the data are stagnant, the corresponding outside world must send out sudden data access and the whole system will have a bottleneck [16, 17]. Based on this, we aim to realize the load balancing of IoT terminal nodes to make the system achieve load balancing and realize the application optimization access function.

Based on the disadvantages of the corresponding data distribution and storage of IoT terminals considering the access cost, this article will propose a complex perceptual data placement algorithm based on the cloud storage distribution of IoT terminal nodes and realize the I/O mode of accurately dividing IoT data by reasonably configuring hybrid perceptual algorithm and adaptive perceptual algorithm to optimize further the performance of the IoT data storage system considering the access cost. Aiming at the problem of terminal node load balancing, this article innovatively proposes the terminal node data sorting and distribution algorithm, which realizes the accurate segmentation of the IoT data to be processed through the node data sorting and distribution algorithm to further accelerate the data reading and processing speed. At the same time, a periodic load balancing algorithm is added to the terminal node data sorting and distribution algorithm so as to further solve the problem of unbalanced node data layout. Based on the proposed algorithm, this article designs a load balancing cloud storage data distribution optimization system of IoT terminal nodes considering access cost and carries out experimental verification in a real environment. The experimental results show that the data pattern division accuracy corresponding to the distribution strategy proposed in this article is improved to 97.13% and the corresponding data access efficiency is improved to 98.3% compared with the traditional distribution strategy. The data distribution strategy proposed in this article has obvious performance advantages and further promotion value.

The main structure of this article is as follows: the second section will mainly analyze the current research status of the load balancing cloud storage data distribution system of IoT terminal nodes considering access cost. The third section will focus on the analysis of complex perceptual data placement algorithm and terminal node data sorting and distribution algorithm and design the cloud storage data distribution optimization system for load balancing of IoT terminal nodes considering access cost. The fourth section will verify the system and analyze its data. Finally, the article is summarized.

## 2. Related Research: Research Status of Load Balancing Cloud Storage Data Distribution System of Internet of Things Terminal Nodes considering Access Cost

A large number of researchers and scientific research institutions have studied and analyzed the data placement and load balancing problems faced by the load of terminal nodes of the IoT. At the research level of data placement and distribution, relevant cloud storage manufacturers in the United States put forward simple storage services, and their corresponding storage mainly involves three indicators: basic storage unit, unique identifier, and storage object container. When there are a few corresponding IoT data, the corresponding distribution strategy is relatively simple; therefore, not too much data will be stored in the corresponding storage object container. However, once there is too much or blocked data, the performance of the cloud storage system will decline, which is not conducive to the maintenance and dynamic development of the system [18, 19]. European cloud storage service providers propose a static directory layering strategy, which mainly adopts a two-tier static object distribution model, alleviating the problem of too centralized data distribution on nodes to a certain extent, but this model still has certain limitations. The even distribution of the corresponding data depends on the corresponding IoT data level [20]. At the level of corresponding load balancing algorithms, relevant Chinese researchers have proposed a scheme to realize load balancing distribution in a distributed environment. However, this scheme does not consider the corresponding data copies, which may lead to multiple data copies appearing on the same data node after the relevant migration. At the same time, this load balancing strategy does not explicitly specify how much data can be migrated during the migration process. If the corresponding migration amount is too large, the load node will exceed the threshold [21]. Relevant European researchers have proposed a load balancing algorithm based on private cloud storage, which mainly realizes the balanced storage of data by calculating the weight corresponding to the storage node. However, the algorithm does not fully consider the heterogeneity of the node itself in the operation process. It treats all nodes equally, and it does not fully consider the heavy data at this time; if the data is too heavy, data migration will lead to the risk of increasing system bandwidth [2, 22]. Based on the analysis of the above research status, the current traditional load data placement algorithm and load balancing algorithm of IoT terminal nodes have more or fewer disadvantages, which leads to the accuracy of data mode division of cloud storage system and then leads to a serious decline in the corresponding data access efficiency.

## 3. Research on Load Balancing Cloud Storage Data Distribution Strategy of Internet of Things Terminal Nodes considering Access Cost

**Figure 1:**
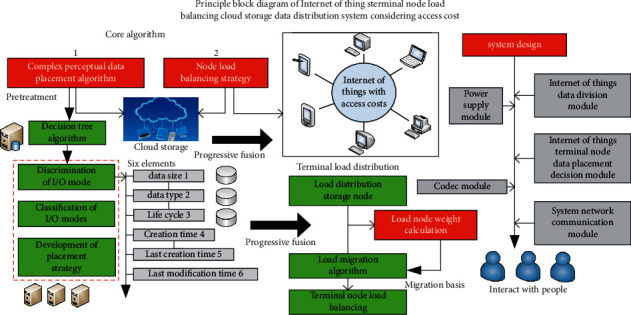
Principle block diagram of Internet of Things terminal node load balancing cloud storage data distribution system considering access cost.

This section mainly analyzes and studies the corresponding key algorithms and system design of the IoT terminal node load balancing cloud storage data distribution system considering access cost. The corresponding system principle block diagram is shown in Figure 1. As shown in Figure 1, the key algorithms proposed in this article mainly have two points: the complex aware data placement algorithm and node load balancing strategy for processing data placement. At the corresponding hardware system design level, it also includes the distribution of IoT terminal nodes, IoT network element data management layer, IoT file service layer, and IoT data node control layer. There is no data management layer in the corresponding system, including IoT user and equipment management, IoT user data node allocation, IoT monitoring data management equipment, and IoT migration data management. The corresponding IoT file service layer includes the upload and download of IoT data files, the upload and download of quantity network audio and video files, the deletion of IoT files and other related modules. The whole system includes monitoring system components.

### 3.1. Complex Perceptual Data Placement Algorithm

**Figure 2:**
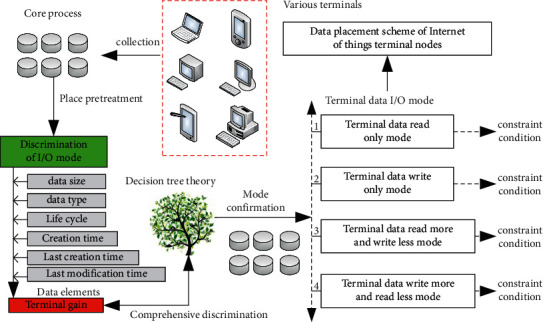
Principle block diagram of complex perceptual data placement algorithm.

The complex perceptual data placement algorithm proposed in this article mainly solves the problem of data placement distribution in the IoT. The complex perceptual data placement algorithm is mainly divided into two processing levels. Firstly, the I/O mode of the terminal data nodes of the IoT is identified and classified based on the decision tree algorithm. Then, the placement strategy is formulated based on the processed IoT data. The principle block diagram of the corresponding complex perceptual data placement algorithm is shown in Figure 2.

The IoT data I/O mode discrimination and classification algorithm based on decision tree mainly discriminates the data I/O type based on the six key factors of IoT data. The corresponding six factors are as follows: IoT data size, data type, IoT data life cycle, IoT data creation time, the last creation time, and the last modification time of IoT data [23–25]. Based on the above six factors, the I/O mode of IoT data is determined. The modes determined in this section are as follows: read-only mode, write-only mode, read more and write less mode, read less and write more mode. The IoT data I/O mode discrimination and classification algorithm based on decision tree mainly follows the following operation steps:

Step 1: calculate the gain value of IoT terminal node data based on six factors: IoT data size, data type, IoT data life cycle, IoT data creation time, IoT data last creation time, and last modification time. The corresponding calculation formulas are shown in formulas (1), (2), and (3), the corresponding IoT data size is *V*, the data type is *L*, the life cycle of IoT data is *S*, the creation time of IoT data is *C*, the last creation time of IoT data is *L*, and the last modification time is *D*.
  (1) $$\begin{array}{c} G\left(\text{data},D\right)=Q\left({\text{data}}_{1}+{\text{data}}_{2}+\cdots +{\text{data}}_{i}\right)-Q\left({\text{data}}_{1}|D_{1}\right)+\cdots Q\left({\text{data}}_{i}|D_{i}\right), \end{array}$$
  (2) $$\begin{array}{c} Q\left(D\right)=-\left[\left.\eta_{1}\left(a_{1}\right)\log\left(\eta_{1}\left(a_{1}\right)\right)\right)+\cdots +\left.\eta_{i}\left(a_{i}\right)\log\left(\eta_{i}\left(a_{i}\right)\right)\right),\right. \end{array}$$
  (3) $$\begin{array}{c} Q\left(\text{data}|D\right)=\eta \left(D_{1}\right)*Q\left({\text{data}}_{1}|D_{1}\right)+\cdots +\eta \left(D_{i}\right)*Q\left({\text{data}}_{i}|D_{i}\right). \end{array}$$
Step 2: divide the I/O mode of the IoT data according to the gain value calculated in Step 1, and repeat the operations of Step 1 and Step 2 for the IoT data until all the IoT data can be defined as unique data mode.
Step 3: generate a data I/O mode classification model of IoT terminal nodes based on a decision tree to determine the complete data I/O type.
Step 4: continuously collect the data to be placed, collect its corresponding six eigenvalues, call the corresponding decision tree IoT terminal node data I/O mode classification model, and constantly put new prediction results into the model so as to improve the accuracy of the model until all IoT terminal node data can be finalized.

After being processed by the I/O mode discrimination and classification algorithm of IoT data in the decision tree, it enters the link of the adaptive data placement algorithm. This algorithm is mainly based on the I/O mode type of IoT data to further determine its corresponding placement strategy and placement scheme. Based on this assumption, the read more write less mode is mode 1, the read less write multimode is mode 2, the read-only mode is mode 3, and the write-only mode is mode 4. Take the read more write less mode and read less write more mode as case modes, and formulate their corresponding placement strategies.

The placement strategy objective function corresponding to the read more write less mode is shown in formula (4). This mode has large downlink traffic and is sensitive to the read delay of the corresponding IoT data. The corresponding data placement constraints in this mode are shown in formulas (5)–(7) and (8). In the corresponding formula, *A* represents the cost proportion threshold and *C* represents the downstream data cost.

(4) $$\begin{array}{c} S=\min\left[\frac{\left(L*G_{1}\left(\text{mode}1\right)\right)}{\left(A*d*w\right)}\right], \end{array}$$

(5) $$\begin{array}{c} L*G_{1}\left(\text{mode}1\right)=\max\frac{\left(C_{1}^{4}*{\mathrm{lg}}_{1}+\cdots +C_{i}^{4}*{\mathrm{lg}}_{i}\right)}{m_{i},\;1<i<n}, \end{array}$$

(6) $$\begin{array}{c} A_{1}=\left(\prod i\in N_{i}\left(1-a_{1}\right)*\prod i\in N_{i}\left(a_{1}\right)+\cdots +\prod i\in N_{i}\left(1-a_{i}\right)*\prod i\in N_{i}\left(a_{i}\right)\right), \end{array}$$

(7) $$\begin{array}{c} A_{1}^{s}+A_{1}^{g}+A_{1}^{T}<a*A_{1}, \end{array}$$

(8) $$\begin{array}{c} A_{1}^{T}=\left(a_{1}^{4}*P_{\eta_{1}}^{T}*G_{d_{1}}\right)+\cdots +\left(a_{1}^{4}*P_{\eta_{i}}^{T}*G_{d_{i}}\right). \end{array}$$

The placement strategy objective function corresponding to the read less write multimode is shown in formula (9). This mode is mainly relatively sensitive to write delay. The corresponding data placement constraints in this mode are shown in formulas (10)–(12) and (13).

(9) $$\begin{array}{c} S=\min\left[\frac{\left(L*G_{2}\left(\text{mode}2\right)\right)}{\left(A*d*w\right)}\right], \end{array}$$

(10) $$\begin{array}{c} L*G_{2}\left(\text{mode}2\right)=\max\frac{\left(C_{1}^{2}*{\mathrm{lg}}_{1}+\cdots +C_{i}^{3}*{\mathrm{lg}}_{i}\right)}{m_{i},\;1<i<n}, \end{array}$$

(11) $$\begin{array}{c} A_{2}=\left(\prod i\in N_{i}\left(1-a_{1}\right)*\prod i\in N_{i}\left(a_{1}\right)+\cdots +\prod i\in N_{i}\left(1-a_{i}\right)*\prod i\in N_{i}\left(a_{i}\right)\right), \end{array}$$

(12) $$\begin{array}{c} A_{2}^{s}+A_{2}^{g}+A_{2}^{T}<a*A_{2}, \end{array}$$

(13) $$\begin{array}{c} A_{2}^{T}=\left(a_{1}^{2}*P_{\eta_{1}}^{T}*G_{d_{1}}\right)+\cdots +\left(a_{1}^{2}*P_{\eta_{i}}^{T}*G_{di}\right). \end{array}$$

Through the confirmation of the above data placement scheme, the data sorting and distribution of IoT terminal nodes can be further confirmed so as to further optimize the data placement of the system.

In summary, based on the above algorithm, the heterogeneity of IoT terminal node data can be fully considered so as to realize the rationality of IOT terminal node data placement considering access cost and then prepare for load balancing.

### 3.2. Terminal Node Load Balancing Strategy

**Figure 3:**
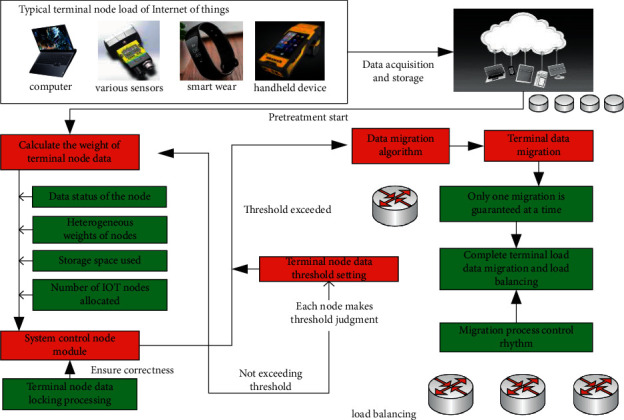
Principle block diagram of terminal node load balancing algorithm.

The terminal node load balancing strategy proposed in this section mainly includes load distribution, storage nodes, and load migration. The principle block diagram of the corresponding load balancing strategy is shown in Figure 3. From Figure 3, we can see the load distribution, storage nodes, and load migration of the entire wireless sensor network.

In data node allocation, the weight of terminal node data needs to be calculated first. The corresponding calculation formula is shown in formula (14). The corresponding *S* represents the data state of the node, the corresponding *a* represents the heterogeneous weight of the data node, *W_s_* represents the storage space used by the corresponding node data, and *W*_*n*_ represents the number of IoT nodes currently allocated. When the corresponding data weight is larger, the comprehensive available resources of the corresponding data node are smaller.

(14) $$\begin{array}{c} W=\frac{\left(S*W_{s}\right)}{\left(W_{T}*A\right)*W_{n}}. \end{array}$$

**Figure 4:**
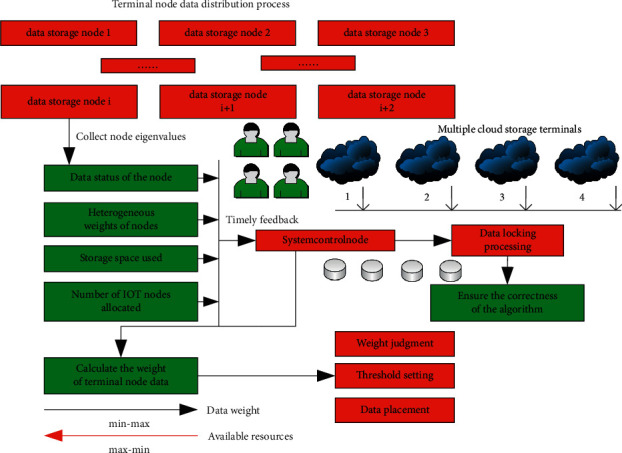
Principle block diagram of terminal node data distribution process.

The corresponding data node allocation process is shown in Figure 4. It can be clearly seen from Figure 4 that each storage node corresponding to the wireless sensor network needs to continuously calculate the heterogeneous weight of its corresponding data node and feed the data back to the corresponding control node of the wireless sensor network in real-time. At the same time, the data also cover key information such as the storage space used by data nodes and the number of IoT nodes allocated. In order to ensure the correct rate of the algorithm of the entire system, the system is implemented with a locking scheme when multiple IoT data nodes are concurrent.

After completing the data node allocation, carry out the data migration operation. First, set the data load threshold as *Wy*, judge whether the load of IoT terminal nodes is too heavy based on this threshold, and carry out relevant migration actions. When the load weight of the corresponding cloud storage system is far less than the load threshold and the weight of the corresponding single load node is greater than the load weight of the whole system, notify the system to trigger the corresponding data migration algorithm for data migration. When the corresponding system load weight is much greater than the load threshold, notify the system to add the corresponding data storage node and notify the system that the load is too heavy at this time. In the actual data migration process, in order to ensure the correctness of data migration, the system needs to ensure that only one data migration operation is allowed at the same time, and the system needs to control the migration rhythm.

### 3.3. Design of Cloud Storage Data Distribution System for Load Balancing of Internet of Things Terminal Nodes Considering Access Cost

**Figure 5:**
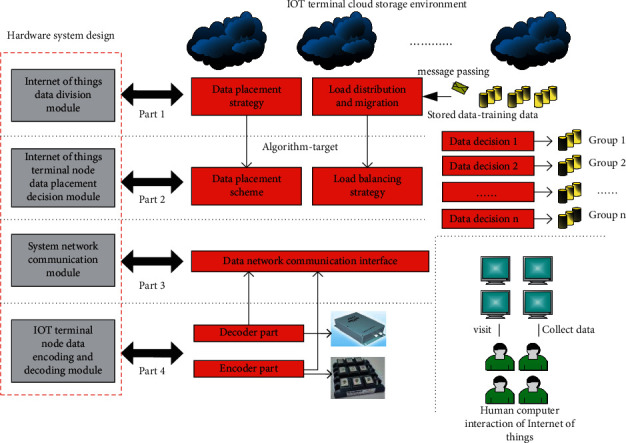
Design framework of cloud storage data distribution system for load balancing of Internet of Things terminal nodes considering access cost.

The design framework of the cloud storage data distribution system for load balancing of IoT terminal nodes considering access cost is shown in Figure 5. It can be seen from the figure that the corresponding hardware system mainly includes IoT data division module, IoT terminal node data placement decision module, system network communication module, and IoT terminal node data encoding and decoding module.

At the design level of the corresponding IoT terminal node data division module, it mainly realizes the I/O mode division of the placed data. The module includes the data I/O model determination strategy, and the corresponding implementation method realizes the data type division model based on the decision tree through scikit-learn. The corresponding data placement decision module of the IoT terminal node is mainly a module to formulate the corresponding data placement strategy, which includes the data placement strategy algorithm. The corresponding network communication module is mainly to realize the interaction between multicloud storage, provide the function of cloud storage to obtain data and services from the upper server, provide the function of independent search and modification and other related operations of the IoT terminal, and provide the function of obtaining and monitoring cloud storage operators. The IoT terminal node data encoding and decoding module mainly helps restore relevant data. This module encodes the IoT terminal data through the encode() function and decodes the relevant IoT terminal data through the decode() function.

## 4. Experimental Verification and Data Analysis

In order to further verify the advantages of the actual system and the corresponding data distribution algorithm, this article is verified by the performance comparison experiment with the traditional distribution algorithm.

**Figure 6:**
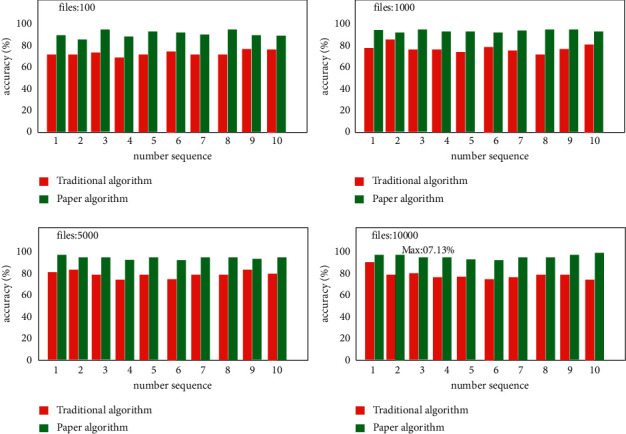
Column chart of data pattern division accuracy experiment.

In the data mode division accuracy experiment, this article selects 100, 1000, 5000, and 10000 IoT terminal node files for comparative experiments. The corresponding experimental data are shown in Figure 6. It can be seen from the figure that the data distribution strategy proposed in this article is improving with the increasing number of files. When the number of files reaches 10000, the corresponding data partition accuracy is improved to 97.13%, which has obvious advantages over the traditional data distribution strategy.

**Figure 7:**
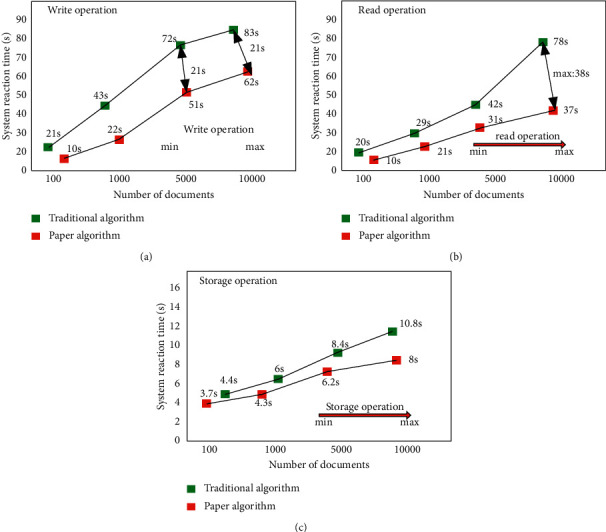
(a) Write operation level performance comparison column chart. (b) Read operation level performance comparison column chart. (c) Storage operation level performance comparison column chart.

Verify the response speed advantages of the distribution algorithm at the read, write, store, and delete levels when the number of files corresponds to 100, 1000, 5000, and 10000. The corresponding experimental results are shown in Figures 7(a), 7(b), and 7(c). As can be seen from the figure, at the corresponding write operation level, the write speed of the algorithm proposed in this article is about 15% higher than that of the traditional algorithm. At the corresponding read operation level, the write speed of the proposed algorithm is about 20% higher than that of the traditional algorithm. At the corresponding storage operation level, the writing speed of the proposed algorithm is about 17% higher than that of the traditional algorithm. At the corresponding level of delete storage operation, the writing speed of the proposed algorithm is about 17% higher than that of the traditional algorithm. Based on the above data, it can be seen that the performance of the algorithm proposed in this article has been comprehensively improved compared with the traditional algorithm at the basic operational level.

**Figure 8:**
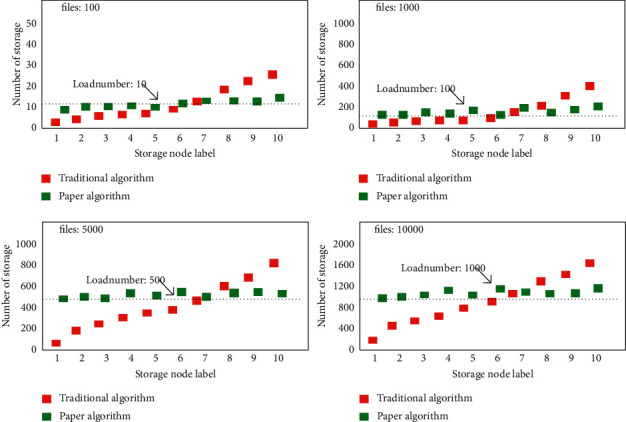
Broken line diagram of load distribution of each storage node under different numbers of files.

In order to verify the advantages of this algorithm in the level of load balancing, the comparative verification is carried out when the number of files of IoT terminal nodes is 100, 1000, 5000, and 10000, and the corresponding load number of each storage node in the system is observed in the experiment. Under different file numbers, the corresponding load distribution is shown in Figure 8. It can be seen from the figure that the load distribution of the distribution algorithm proposed in this article is relatively uniform in each memory. At the same time, with the increase of the number of files, the number of files between the corresponding memories fluctuates little, while the corresponding load is seriously unbalanced under the corresponding traditional distribution strategy.

**Figure 9:**
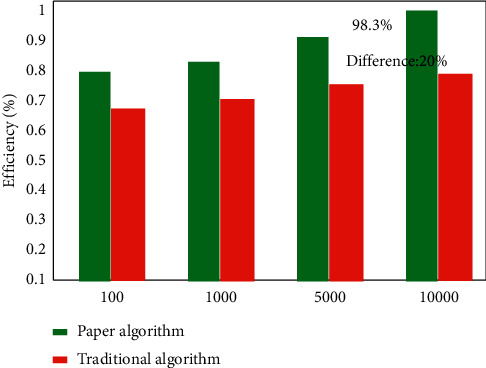
Comparison of system data access efficiency under different numbers of files.

In order to verify the superiority of the algorithm proposed in this article in system data access efficiency, a comparative experiment is carried out based on the traditional distribution strategy. The experimental results are shown in Figure 9. It can be seen from the figure that the distribution strategy proposed in this article has the highest system data access efficiency of 98.3%, which is nearly 20% higher than the traditional distribution algorithm. Therefore, this algorithm has obvious advantages over traditional algorithms.

The above analysis and experimental results further show that the algorithm proposed in this article has obvious advantages over the traditional algorithm in the cloud storage data system of load balancing of IoT terminal nodes considering access cost, and it can realize the optimization of data placement and load balancing. This greatly improves the accuracy of the system's data mode division of IoT terminal nodes and further improves the data access efficiency of the system.

## 5. Conclusion

This article mainly analyzes and studies the relevant research algorithms of cloud storage data distribution system with load balancing of IoT terminal nodes considering access cost and analyzes and compares their advantages and disadvantages. Based on the existing problems, this article proposes a complex sensing data placement algorithm based on the cloud storage distribution of IoT terminal nodes and realizes the accurate division of I/O mode of IoT data by reasonably configuring hybrid sensing algorithm and adaptive sensing algorithm so as to further optimize the performance of IoT data storage system considering access cost. Aiming at the problem of terminal node load balancing, this article innovatively proposes the terminal node data sorting and distribution algorithm. Through the node data sorting and distribution algorithm, the accurate segmentation of the IoT data to be processed is realized so as to further accelerate the data reading and processing speed. At the same time, a periodic load balancing algorithm is added to the terminal node data sorting and distribution algorithm. Thus, the problem of unbalanced node data layout is further solved. Based on the proposed algorithm, this article designs a load balancing cloud storage data distribution optimization system of IoT terminal nodes considering access cost and carries out experimental verification in a real environment. The experimental results show that the data pattern division accuracy corresponding to the distribution strategy proposed in this article is improved to 97.13% and the corresponding data access efficiency is improved to 98.3%, compared with the traditional distribution strategy. The data distribution strategy proposed in this article has obvious performance advantages and further promotion value. In the follow-up research, the article will focus on analyzing the energy consumption of wireless sensor networks. At the same time, it will further optimize the load balancing situation in the load balancing strategy. Moreover, it will further discuss the load balancing strategy problem of the traditional cloud storage network considering the cost.

## Data Availability

The data used to support the study are available from the corresponding author upon request.
